# Life satisfaction mediates advanced maternal age at childbirth and frailty across cohorts

**DOI:** 10.1016/j.isci.2026.116023

**Published:** 2026-05-22

**Authors:** Zheng-Hui Zhao, Wenze Tian, Xiaoyu Wang

**Affiliations:** 1 Guangzhou Key Laboratory of Metabolic Diseases and Reproductive Health, Guangdong-Hong Kong Metabolism & Reproduction Joint Laboratory, Reproductive Medicine Center, The Affiliated Guangdong Second Provincial General Hospital of Jinan University, Guangzhou 510317, China; 2 School of Sociology, Nankai University, Tianjin 300353, China; 3 School of Public Administration, South China Agricultural University, Guangzhou 510642, China

**Keywords:** Health sciences, Internal medicine, Life style, Medicine, Mental state, Patient social context, Pregnancy, Psychosocial factor, Public health, Women’s health

## Abstract

Advanced maternal age at childbirth (AMAC) is increasingly common, yet its long-term association with frailty remains unclear. Using three longitudinal cohorts (CHARLS, KLoSA, and HRS; *N* = 21,090), we examined this association via Cox proportional hazards models and mediation analysis. After multivariable adjustment, AMAC (≥35 years) was associated with increased frailty risk in the Chinese (hazard ratio [HR]: 1.21, 95% confidence interval [CI]: 1.03–1.42) and Korean (HR: 1.24, 95% CI: 1.02–1.50) cohorts, while AMAC (≥40 years) showed a similar direction in the United States cohort. Life satisfaction partially mediated this association, accounting for 8.9% of the total effect in KLoSA and 22.4% in HRS, with substantial attenuation of the direct effect in CHARLS. These cross-national findings indicate that psychosocial well-being may represent a modifiable pathway for frailty prevention among women with a history of AMAC.

## Highlights

• AMAC is associated with elevated frailty risk in later life
• Life satisfaction partially mediates the AMAC-frailty association
• The AMAC-frailty association varied across behavioral subgroups

## Graphical abstract

**Figure:**
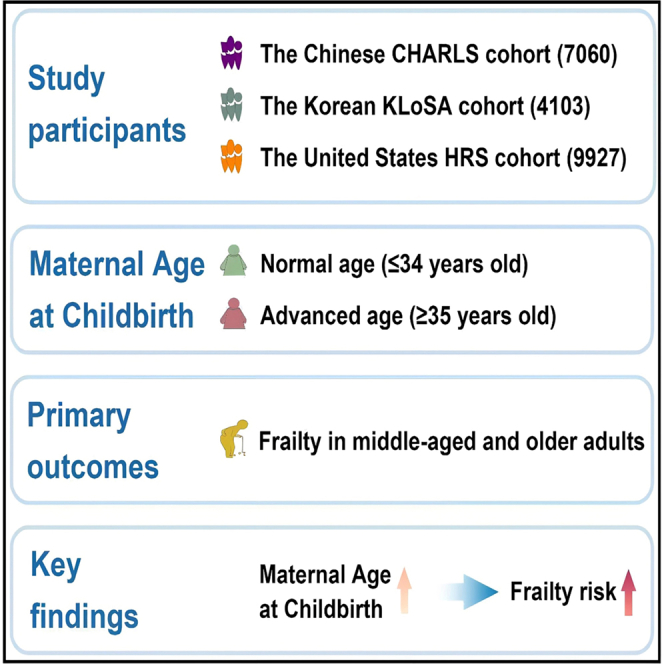

## Introduction

Frailty has emerged as a major public health challenge amid rapid population aging. It reflects heightened vulnerability and impaired recovery from stressors and is consistently linked to adverse outcomes, including falls, disability, and incident chronic diseases.^1^^,^^2^ At the molecular level, frailty is frequently accompanied by chronic inflammation and impaired autonomic function, which contribute to endothelial dysfunction and an elevated risk of cardiovascular events.^3^^,^^4^ Moreover, at the behavioral level, frailty is often associated with lifestyle alterations, including diminished physical activity and greater social isolation.^5^ Optimizing early frailty assessment and implementing individualized prevention strategies may therefore represent a key leverage point for improving the management of complex multimorbidity. Notably, frailty is more prevalent among women than men,^6^ suggesting that female reproductive factors may play a particularly important role in shaping frailty risk. Frailty can be conceptualized through two primary frameworks: the Fried frailty phenotype, which identifies frailty as a clinical syndrome characterized by five physical criteria (weakness, slowness, low activity, exhaustion, and shrinking), and the deficit accumulation model, which quantifies frailty as the proportion of health deficits present from a comprehensive list. The present study adopts the deficit accumulation approach via the frailty index (FI), which captures a broader spectrum of health deficits across multiple domains and is well suited for secondary analysis of large-scale survey data.^7^

The prevalence of AMAC has been steadily rising worldwide in recent decades.^8^ This trend is largely driven by socioeconomic shifts, including delayed marriage, increased pursuit of advanced education and careers, and the development of assisted reproductive technologies.^9^^,^^10^ In general, AMAC is commonly defined as pregnancy at or after 35 years, a threshold widely adopted in many literatures.^11^ Although postponing childbearing may confer certain advantages, such as enhanced socioeconomic stability, emotional readiness, and improved educational outcomes for offspring,^12^ maternal age remains an independent risk factor for a range of adverse perinatal and obstetric outcomes. These include fetal growth restriction, preterm delivery, preeclampsia, gestational diabetes mellitus, and postpartum depression.^12^^,^^13^^,^^14^ However, despite growing recognition of AMAC as a significant public health concern, evidence regarding the long-term health consequences for mothers beyond the perinatal window remains strikingly limited. Although several studies have explored the associations between female reproductive factors and frailty, including age at menarche and menopause, age at first sexual intercourse, age at first birth, and history of pregnancy loss,^15^^,^^16^ the mechanisms underlying the relationship between pregnancy in women of advanced maternal age and frailty remain poorly understood. Therefore, longitudinal studies are urgently needed to examine how AMAC shapes women’s frailty risk in middle and later life.

The concept of life satisfaction refers to an individual’s overall cognitive assessment of their life status. Multiple studies have demonstrated that elevated life satisfaction protects against frailty, whereas the emergence of frailty frequently coincides with deteriorations in this domain.^17^^,^^18^ Additionally, life satisfaction is significantly lower in women of advanced age than in younger women, driven by heightened psychological distress, an elevated prevalence of adverse pregnancy outcomes, and underlying socioeconomic challenges.^19^^,^^20^^,^^21^^,^^22^^,^^23^ These findings suggest that life satisfaction potentially functions as a mediating mechanism linking AMAC to frailty. Therefore, leveraging longitudinal data to disentangle this temporal relationship is essential for formulating highly targeted preventive strategies.

Building on this background, the present study aims to elucidate the dynamic association between AMAC and frailty, with a focus on uncovering the social and psychosocial pathways that may underlie this relationship. We utilize three large-scale longitudinal national cohorts (China Health and Retirement Longitudinal Study [CHARLS]; Korean Longitudinal Study of Aging [KLoSA]; Health and Retirement Study [HRS]), which encompass diverse ethnic, cultural, and socioeconomic contexts, thereby enhancing the generalizability of our findings and enabling comparative analyses of potential population-specific differences in the AMAC-frailty association. Using Cox proportional hazards models, mediation mechanism test, and subgroup analysis, we move beyond traditional regression approaches to capture the temporal and mechanistic complexity of this interplay. Our findings provide preliminary but meaningful insights into how AMAC may influence frailty risk in later life, offering a foundation for future research and targeted interventions aimed at mitigating long-term health risks among women with advanced maternal age pregnancies.

## Results

### Baseline characteristics

**Figure 1:**
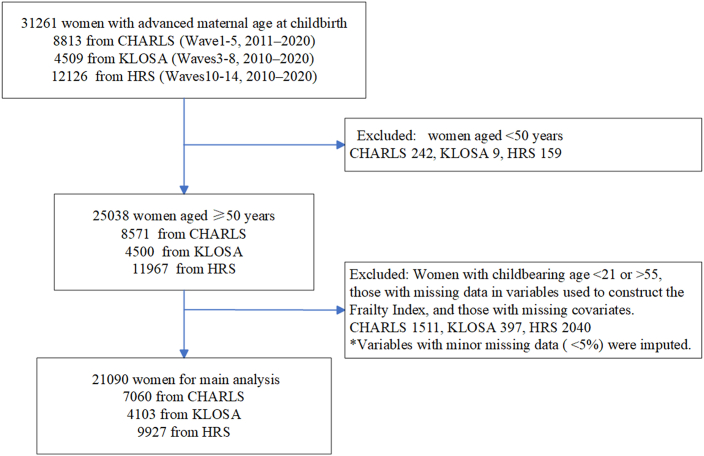
The flowcharts of baseline participant selection in CHARLS, KLoSA, and HRS

The study included 7,060 participants from CHARLS, 4,103 participants from KLoSA, and 9,927 participants from HRS (Figure 1). Table 1 presents baseline characteristics across the three cohorts. The proportion of women with AMAC (≥35 years) ranged from 14.45% in CHARLS and 17.94% in KLoSA to 42.95% in HRS. The average age was 59.59 years in CHARLS, 65.41 in KLoSA, and 66.23 in HRS. Educational attainment was markedly lower in CHARLS and KLoSA, where 74.27% and 73.92% of participants, respectively, had not completed high school, compared with only 18.94% in the HRS cohort, where a substantially larger proportion had finished high school. Lifestyle factors also varied markedly across cohorts. The proportion of current smoking was 8.12% in CHARLS, 4.41% in KLoSA, and 48.94% in HRS, while current drinking was reported by 17.45% in CHARLS, 98.05% in KLoSA, and 49.54% in HRS. Social activity and physical activity were more frequently reported in CHARLS than in KLoSA and HRS. Additionally, we compared the differences in key variables between the included and excluded populations across the three databases (Tables S2–S4).

**Table 1:** Baseline characteristics of participants by AMAC status in CHARLS, KLoSA, and HRS

Characteristics	CHARLS *N* (%)	*p* value	KLoSA *N* (%)	*p* value	HRS *N* (%)	*p* value
Total	7,060	–	4,103	–	9,927	–
Maternal age, *n* (%)						
<35	6,040 (85.55 )	–	3,367 (82.06)	–	5,663 (57.05)	–
≥35	1,020 (14.45 )	–	736 (17.94)	–	4,264 (42.95)	–
Age, mean (SD)	59.59 (8.45)	<0.001	65.41 (10.74)	<0.001	66.23 (11.66)	0.007
Education, *n* (%)	–	<0.001	–	<0.001	–	<0.001
Below high school	5,241 (74.27)	–	3,033 (73.92)	–	1,880 (18.94)	–
High school	1,170 (16.58)	–	880 (21.45)	–	6,209 (62.55)	–
Above high school	646 (9.15)	–	190 (4.63)	–	1,838 (18.52)	–
Marital status, *n* (%)	–	<0.001	–	<0.001	–	<0.001
Unmarried and others	1,038 (14.70)	–	1,360 (33.15)	–	4,399 (44.31)	–
Married and partnered	6,022 (85.30)	–	2,743 (66.85)	–	5,528 (55.69)	–
Smoking, *n* (%)	–	0.003	–	<0.001	–	0.892
No	6,487 (91.88)	–	3,922 (95.59)	–	5,069 (51.06)	–
Yes	573 (8.12)	–	181 (4.41)	–	4,858 (48.94)	–
Drinking, *n* (%)	–	0.285	–	0.050	–	<0.001
No	5,828 (82.55)	–	80 (1.95)	–	5,009 (50.46)	–
Yes	1,232 (17.45)	–	4,023 (98.05)	–	4,918 (49.54)	–
Employment, *n* (%)	–	<0.001	–	<0.001	–	<0.001
Unemployed	930 (13.17)	–	937 (22.87)	–	3,951 (39.80)	–
Working or retired	6,130 (86.83)	–	3,160 (77.13)	–	5,976 (60.20)	–
Social activity, *n* (%)	–	0.020	–	0.100	–	<0.001
No	3,405 (47.86)	–	2,743 (66.85)	–	6,815 (68.65)	–
Yes	3,710 (52.14)	–	1,360 (33.15)	–	3,112 (31.35)	–
Physical activity, *n* (%)	–	<0.001	–	<0.001	–	<0.001
No	3,383 (47.92)	–	2,833 (69.05)	–	6,220 (62.66)	–
Yes	3,677 (52.08)	–	1,270 (30.95)	–	3,707 (37.34)	–
Personal income (logarithm), mean (SD)	10.37 (1.64)	<0.001	1.13 (2.52)	<0.001	3.60 (4.85)	<0.001

*p* values were calculated to compare baseline characteristics between the AMAC and non-AMAC groups using Student’s *t* tests for continuous variables and Pearson’s chi-square tests for categorical variables. Maternal age group was the exposure definition and was therefore not tested.

### Association between AMAC and frailty

**Figure 2:**
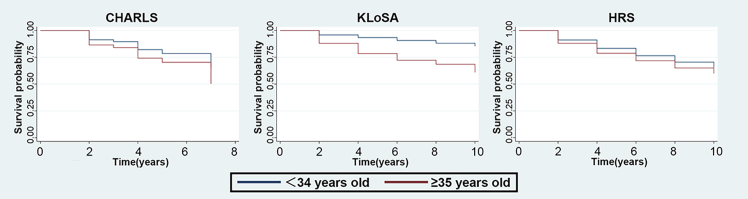
Kaplan-Meier survival curves of frailty risk based on AMAC (<35 years vs. ≥35 years) in CHARLS, KLoSA, and HRS Frailty risk: frailty status (non-frail or frail). Survival probability diverged early and persisted throughout follow-up, with the greatest separation observed in KLoSA and a modest yet consistent difference in HRS. Log rank test results across all three datasets indicate significant differences between groups (*p* < 0.001).

Kaplan-Meier analysis showed a consistently higher cumulative risk of frailty among women with AMAC compared with those without AMAC (Figure 2). Across CHARLS, KLoSA, and HRS, women with an advanced maternal age at last childbirth (≥35 years) consistently exhibited lower frailty-free survival than those whose last childbirth occurred before age 35. The separation between the survival functions emerged early and persisted throughout follow-up, being most pronounced in KLoSA and modest yet directionally consistent in HRS. Log rank tests indicated significant differences in frailty-free survival between groups (*p* < 0.001).

Additionally, we conducted Cox regression analysis to further explore the relationship between baseline AMAC and frailty risk (Table 2). Results showed that AMAC was associated with elevated frailty risk in minimally adjusted analyses, with attenuation after progressive covariate adjustment. In the fully adjusted model (model 4), AMAC remained associated with higher frailty risk in CHARLS (hazard ratio [HR]: 1.21, 95% confidence interval [CI]: 1.03–1.42; *p* = 0.019) and KLoSA (HR: 1.24, 95% CI: 1.02–1.50; *p* = 0.032). In HRS, the point estimate was in the same direction but did not reach statistical significance (HR: 1.10, 95% CI: 0.99–1.22; *p* = 0.063).

**Table 2:** Hazard ratios (95% CIs) for the association of AMAC with incident frailty

	CHARLS	*p*	KLoSA	*p*	HRS^a^	*p*
	HR (95% CI)		HR (95% CI)		HR (95% CI)	
Model 1	1.52 (1.31, 1.76)	<0.001	3.03 (2.54, 3.61)	<0.001	1.22 (1.10, 1.35)	<0.001
Model 2	1.24 (1.05, 1.45)	0.009	1.33 (1.10, 1.62)	0.004	1.13 (1.02, 1.25)	0.016
Model 3	1.23 (1.05, 1.44)	0.012	1.32 (1.09, 1.60)	0.005	1.13 (1.02, 1.25)	0.022
Model 4	1.21 (1.03, 1.42)	0.019	1.24 (1.02, 1.50)	0.032	1.10 (0.99, 1.22)	0.063

Model 1 included no covariates; model 2 was adjusted for age, education, and marital status; model 3 further adjusted smoking and drinking; model 4 was fully adjusted, further controlling employment, social activity, physical activity, and natural logarithm of personal income.

a In the HRS data, AMAC was defined using a cutoff of 40 years of age for Cox regression analysis.

The proportional hazards assumption was satisfied for CHARLS (global test *p* = 0.620) and HRS (global test *p* = 0.762). For KLoSA, the global test was significant (*p* = 0.039), primarily driven by the retirement variable (*p* = 0.019). However, the main exposure variable (AMAC) showed no evidence of non-proportionality (*p* = 0.759), supporting the validity of the primary findings (Table S5).

### Non-linear relationship between AMAC and frailty

**Figure 3:**
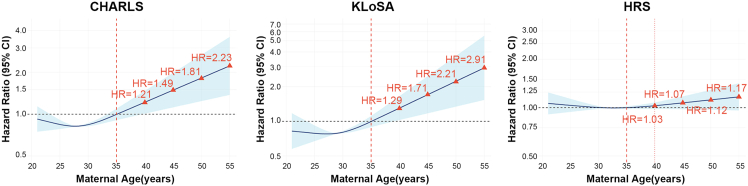
RCS curves of the relationship between maternal age and frailty risk in CHARLS, KLoSA, and HRS Frailty risk: frailty index. The solid line represents the estimated hazard ratio (HR), and the shaded area represents the 95% confidence interval (CI). All analyses have been adjusted for age, education, marital status, employment, smoking, drinking, social activity, physical activity, and personal income (logarithm). The dashed horizontal line at *y* = 1 denotes the baseline HR (HR = 1).

Restricted cubic spline (RCS) models, adjusted for full covariates (model 4), revealed a clear dose-response pattern in CHARLS and KLoSA (Figure 3). In CHARLS, there was strong evidence for an overall association (*p* for overall <0.001) and a statistically significant non-linear relationship (*p* for non-linear = 0.010), with the curve remaining relatively flat at younger ages and then increasing more steeply after 35 years, indicating a higher frailty hazard at older maternal ages.

In KLoSA, the overall association was also significant (*p* for overall <0.001), while evidence for non-linear relationship was weaker (*p* for non-linear = 0.064), consistent with an approximately monotonic increase in frailty risk as maternal age increased, particularly beyond 35 years. In contrast, the HRS spline curve was comparatively flat, with no evidence supporting either an overall association (*p* for overall = 0.289) or a non-linear relationship (*p* for non-linear = 0.204), suggesting that maternal age at last childbirth showed a limited dose-response association with incident frailty in this cohort (Figure 3).

### The ability of AMAC parameters to predict the risk of frailty

**Figure 4:**
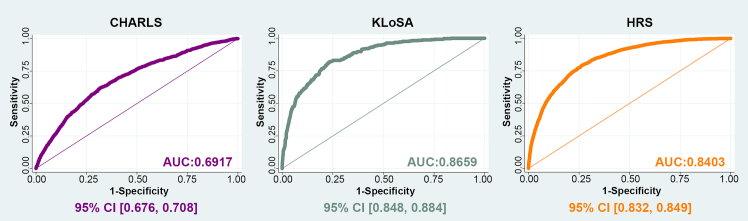
ROC curves for frailty risk in CHARLS, KLoSA, and HRS Frailty risk: frailty status. Data are presented as area under the curve (AUC) with 95% confidence interval (CI). All analyses have been adjusted for age, education, marital status, employment, smoking, drinking, social activity, physical activity, and personal income (logarithm).

To compare the predictive performance, receiver operating characteristic (ROC) curves were generated for each Cox regression model (Figure 4). The results indicated different performance across cohorts: the area under the curve (AUC) was 0.6917 (95% CI: 0.676–0.708) in CHARLS, 0.8659 (95% CI: 0.848–0.884) in KLoSA, and 0.8403 (95% CI: 0.832–0.849) in HRS. Overall, these results indicate moderate performance in CHARLS and outperformed ability in KLoSA and HRS, with the strongest predictive performance observed in KLoSA (Figure 4). To further evaluate the incremental discriminatory value of AMAC, we conducted an additional analysis comparing the adjusted ROC model with a covariate-only model that excluded AMAC. The results showed negligible differences in AUC across cohorts (ΔAUC = 0.0007 in CHARLS, 0.0001 in HRS, and −0.0001 in KLoSA), with no changes reaching statistical significance (Table S6).

### Mediation analysis

**Figure 5:**
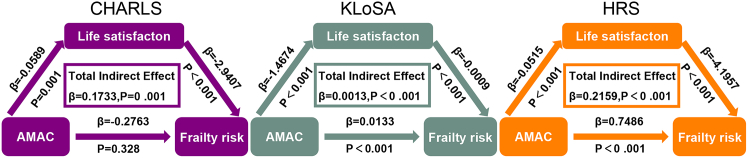
Association between AMAC, life satisfaction, and frailty risk Frailty risk: frailty index. All analyses have been adjusted for age, education, marital status, employment, smoking, drinking, social activity, physical activity, and personal income (logarithm).

We performed mediation analysis to investigate the potential role of life satisfaction in the relationship between AMAC and frailty risk. Mediation analyses revealed that life satisfaction partially mediated the association between AMAC and frailty, with cohort-specific patterns (Figure 5). In CHARLS, AMAC was negatively associated with life satisfaction (β = −0.059, *p* = 0.001), and life satisfaction was negatively associated with frailty (β = −2.941, *p* < 0.001). The indirect effect of AMAC on frailty through life satisfaction was statistically significant (indirect effect = 0.173, *p* = 0.001). The direct effect of AMAC on frailty was not significant after accounting for life satisfaction (β = −0.276, *p* = 0.328), suggesting potential full mediation. However, given that the direct and indirect effects were in opposite directions (indicating inconsistent mediation), the proportion mediated was not calculated; instead, we focused on the significance of the indirect pathway.

In KLoSA, the indirect effect was smaller but statistically significant (indirect effect = 0.089, *p* = 0.024), with 8.9% of the total effect mediated by life satisfaction. In HRS, the indirect effect was more pronounced (indirect effect = 0.224, *p* < 0.001), with 22.4% of the total effect mediated. These findings suggest that life satisfaction plays a consistent mediating role across cohorts, although the magnitude varies by population context.

### Subgroup analysis

**Figure 6:**
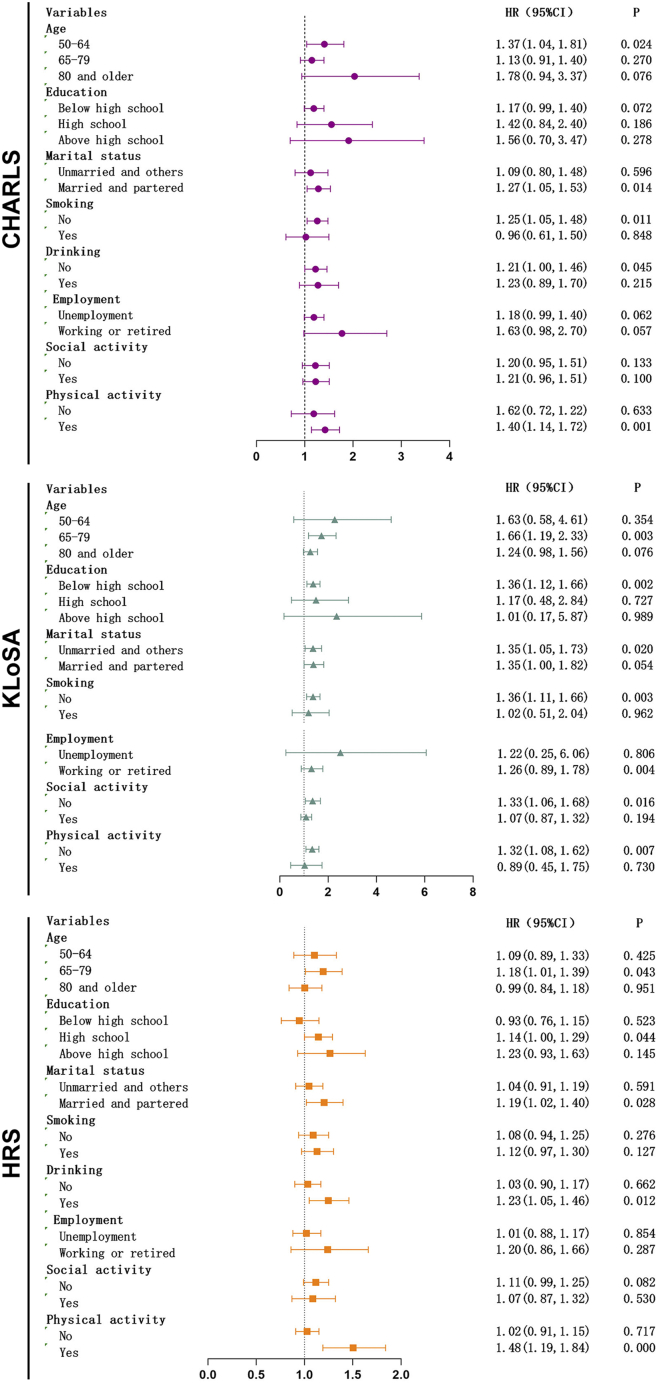
Association between AMAC with frailty risk across subgroups Frailty risk: frailty status (non-frail or frail). Data are presented as hazard ratios (HRs) with 95% confidence interval (CI); the point estimate is shown as a square, and horizontal lines represent the 95% CI. The number of frailty events was insufficient in the drinking subgroup analysis of the KLoSA dataset, so the analysis was skipped automatically.

Subgroup analyses were conducted to describe the AMAC-frailty association across strata of age, education, marital status, smoking, drinking, employment, social activity, and physical activity (Figure 6). Overall, point estimates were generally above unity across most strata, although statistical significance varied by subgroup, likely reflecting differences in precision and event numbers.

In CHARLS, the association was strongest among women aged 50–64 years (HR: 1.37, 95% CI: 1.04–1.81) and those who were married/partnered (HR: 1.27, 95% CI: 1.05–1.53). Significant associations were also observed among non-smokers (HR: 1.25, 95% CI: 1.05–1.48), non-drinkers (HR: 1.21, 95% CI: 1.00–1.46), and physically active women (HR: 1.40, 95% CI: 1.14–1.72). No significant associations were detected in the corresponding opposite strata. Educational level, employment, and social activity did not materially modify the association.

In KLoSA, the association was most pronounced among women aged 65–79 years (HR: 1.66, 95% CI: 1.19–2.33) and those with less than a high school education (HR: 1.36, 95% CI: 1.12–1.66). Significant associations were also observed among non-smokers (HR: 1.36, 95% CI: 1.11–1.66), women without social activity (HR: 1.33, 95% CI: 1.06–1.68), and inactive women (HR: 1.32, 95% CI: 1.08–1.62). Estimates for the opposite strata were consistently non-significant. Marital status and employment showed no substantial differences.

In HRS, the association was strongest among women aged 65–79 years (HR: 1.18, 95% CI: 1.01–1.39) and those who were married/partnered (HR: 1.19, 95% CI: 1.02–1.40). Notable effect modification was observed for drinking and physical activity: the association was significant among drinkers (HR: 1.23, 95% CI: 1.05–1.46) but not non-drinkers and among physically active women (HR: 1.48, 95% CI: 1.19–1.84) but not inactive women. Education, smoking, employment, and social activity did not materially modify the association.

### Sensitivity analyses

We conducted several sensitivity analyses to assess the robustness of our findings. These analyses collectively help to determine the robustness of the primary findings across different exposure definitions, additional confounders, time periods, and population subgroups.

First, to evaluate whether the association between AMAC and frailty was sensitive to the choice of exposure cutoff, we repeated the main analysis using a five-category classification of age at last childbirth (<25, 25–29, 30–34 [reference], 35–39, and ≥40 years) (Table S7). Overall, estimates were directionally consistent across cohorts and generally attenuated with sequential adjustment. Focusing on the fully adjusted model (model 4), in CHARLS, women with age at last childbirth ≥35 years tended to have higher frailty risk than the reference group, although the estimates were less precise and the joint test for the five-category exposure did not provide strong evidence of overall differences (Wald *p* = 0.205; midpoint-based *p* trend = 0.113). In KLoSA, the five-category specification remained associated with incident frailty in model 4 (Wald *p* = 0.046), accompanied by a modest monotonic pattern in the midpoint-based trend test (*p* trend = 0.040). In HRS, the elevated risk for age at last childbirth ≥40 years persisted after full adjustment (HR: 1.17, 95% CI: 1.03–1.32; *p* = 0.013), whereas evidence for differences across all five categories was not statistically significant (Wald *p* = 0.126; *p* trend = 0.560). Overall, results were robust to alternative exposure categorization.

In addition, Kaplan-Meier curves stratified by the five categories of age at last childbirth (Figure S2) showed a clear separation of frailty-free survival functions across exposure groups, with the lowest frailty-free survival observed in the oldest age-at-last-childbirth category. Differences between curves were statistically significant in each cohort (log rank *p* < 0.001).

Second, to address potential confounding by additional reproductive factors not included in the main models, we further adjusted for parity (model 5) in the fully adjusted Cox models, where data were available (Table S8).

Third, to examine whether the observed associations were influenced by the COVID-19 pandemic, we conducted an analysis excluding all follow-up data from the year 2020. As shown in Table S9, the overall pattern of associations remained largely unchanged. In CHARLS, AMAC remained significantly associated with incident frailty in the fully adjusted model (HR = 1.20, 95% CI: 1.02–1.41, *p* = 0.024). In KLoSA and HRS, the associations were significant in the crude and partially adjusted models, whereas the fully adjusted estimates were attenuated and no longer statistically significant (KLoSA: HR = 1.18, 95% CI: 0.96–1.55, *p* = 0.121; HRS: HR = 1.10, 95% CI: 0.99–1.23, *p* = 0.076). Nevertheless, the effect estimates in all three cohorts remained in the same direction as those in the primary analyses. Consistent with the Cox regression results, Kaplan-Meier curves based on the pre-2020 follow-up data continued to show lower frailty-free survival probabilities among women with AMAC across the three cohorts (Figure S3).

Fourth, to explore potential effect modification and identify whether the AMAC-frailty association varies across key sociodemographic and behavioral characteristics, we performed subgroup analyses and formally tested interactions by including AMAC × subgroup interaction terms in the fully adjusted models (Tables S10–S12). In CHARLS, no interaction was statistically significant (all *p* interaction >0.05). Estimates were higher in some strata (e.g., age 50–64 years: HR = 1.37, 95% CI: 1.04–1.80; non-smokers: HR = 1.25, 95% CI: 1.06–1.49; physically active: HR = 1.32, 95% CI: 1.05–1.67), but interactions for education, marital status, and drinking were not significant (*p* interaction = 0.616, 0.211, and 0.800). In KLoSA, interaction tests were also non-significant across examined factors (all *p* interaction >0.05), and subgroup estimates were less precise in smaller strata (e.g., above high school education: HR = 1.91, 95% CI: 0.85–4.31). In HRS, evidence of interaction was observed for drinking (*p* interaction = 0.042) and physical activity (*p* interaction = 0.002). AMAC was associated with higher frailty risk among drinkers (HR = 1.27, 95% CI: 1.01–1.59) but not among non-drinkers (HR = 1.02, 95% CI: 0.89–1.16), and the association was stronger among physically active women (HR = 1.48, 95% CI: 1.23–1.79) than among inactive women (HR = 1.02, 95% CI: 0.91–1.14). A borderline interaction was noted for marital status (*p* interaction = 0.071), whereas interactions for age, education, smoking, employment, and social activity were not significant (all *p* interaction >0.05).

## Discussion

This study investigated the association between AMAC and frailty using data from three international longitudinal databases, including CHARLS, KLoSA, and HRS. The results showed that AMAC significantly increased the risk of frailty among middle-aged and older adults, with life satisfaction acting as a mediator. These associations were generally consistent in direction across the examined subgroups.

In addition, the robustness of our findings was supported by several sensitivity analyses. Reclassifying maternal age at last childbirth into five categories yielded directionally similar results across cohorts, and interaction analyses suggested that the overall association was generally stable across most examined subgroups. Because follow-up in all three cohorts extended into 2020, the COVID-19 pandemic may also have influenced frailty progression through multiple pathways, including reduced healthcare access, changes in physical activity and social participation, and increased psychosocial stress. To address this concern, we conducted additional analyses excluding the 2020 data. The overall pattern of associations remained broadly consistent with the primary findings, and Kaplan-Meier curves based on the pre-2020 follow-up data showed a similar pattern across the three cohorts. Together, these results support the robustness of the observed association and suggest that it was unlikely to be solely driven by model specification, subgroup composition, or the pandemic-period follow-up.

By confirming the AMAC-frailty association across countries and elucidating the mediating role of life satisfaction, this study provides evidence to inform tailored public health strategies aimed at improving older adults’ health outcomes. These findings underscore the importance of life satisfaction for interventions with significant clinical potential.

This study further highlights the relationship between reproduction factors and frailty. For instance, several studies found that having more children was associated with a higher risk of frailty, suggesting a potential cumulative burden of childbearing on maternal health, especially in later life.^24^^,^^25^^,^^26^ However, other studies report an inverse relationship, where having more children is associated with a lower risk of frailty, possibly reflecting protective social and psychological factors such as increased family support or a stronger sense of purpose.^27^^,^^28^ These contrasting findings may be attributed to differences in study design, sample characteristics, and the inclusion of confounding variables, such as socioeconomic status, social support, and health behaviors. Moreover, both earlier and later menarche ages have been associated with an increased risk of frailty.^24^^,^^28^^,^^29^ Furthermore, several studies have shown that earlier menopause was also associated with a higher risk of frailty.^28^^,^^29^^,^^30^

In addition, age at births may serve as a long-term determinant of frailty in later life. For example, several studies have shown that early birth age is significantly associated with the likelihood of frailty among middle-aged and older women.^28^^,^^31^^,^^32^ Contrary to these studies, one study from China showed that there was no significant association between age at first livebirth and frailty.^24^ One potential explanation for this discrepancy is that the Chinese study used cross-sectional data, which capture information at a single point in time, whereas the other studies employed longitudinal cohort designs that track participants over an extended period. There has been no research on the relationship between AMAC and frailty. Our study found that AMAC was associated with higher odds of frailty among older adults in China, South Korea, and the United States. Women who give birth at older ages often coincide with accelerated reproductive aging, which may reflect or contribute to systemic biological aging processes. Additionally, AMAC is sometimes linked to higher rates of pregnancy complications, which can have long-term metabolic and cardiovascular consequences that increase vulnerability to frailty decades later.

As life expectancy increases, identifying protective factors against adverse health events becomes increasingly important. Recently, life satisfaction has been shown to be a protective factor against adverse health events in older adults.^17^ In this study, our mediation analyses suggest that life satisfaction plays a mediating role in the association between AMAC and frailty. Notably, the mediation signal was strongest in China, where the direct AMAC-frailty association was largely attenuated after accounting for life satisfaction, whereas mediation was partial in Korea (8.9% mediated) and the United States (22.38% mediated). This pattern suggests that psychosocial well-being plays a central role in shaping the long-term health consequences of late-life childbearing, especially within the Chinese sociocultural context. Our results showed that AMAC was negatively associated with life satisfaction, and life satisfaction was also negatively associated with frailty, which was similar to the previous study.^33^ A plausible explanation for our results is that women who give birth at an advanced age may experience prolonged parenting responsibilities into older ages, leaving fewer opportunities to invest in their own health. Lower life satisfaction may then contribute to frailty through behavioral pathways. Mechanistically, affective well-being, particularly life satisfaction, may link AMAC to later life frailty. Clinically, brief screening for low life satisfaction or depressive symptoms could identify at-risk women for early intervention. As a modifiable factor, affective well-being warrants evaluation as a target for frailty prevention in this population.

Nevertheless, the association between AMAC and frailty is presumably multifactorial, arising from a complex interplay of biological, physiological, and socioeconomic determinants. Biologically, pregnancies at advanced age are associated with a significantly elevated risk of gestational diabetes and hypertension compared to those in younger women.^9^^,^^34^ These adverse obstetric outcomes can induce enduring metabolic and cardiovascular alterations, thereby predisposing individuals to frailty decades later. Psychosocially, life satisfaction appears to be related to depressive symptoms,^35^ and depressive symptoms often coexist with frailty in middle-aged and older adults.^36^ Notably, mothers of advanced age have been observed to exhibit lower levels of life satisfaction relative to their younger counterparts.^19^ Finally, socioeconomic factors appear to play a critical moderating role in the AMAC-frailty link. For example, unemployment and financial stressors are usually associated with low life satisfaction.^37^^,^^38^ Women with AMAC who have higher education and income may be better able to buffer against frailty through improved access to healthcare, nutrition, and other health-promoting resources. By contrast, in some contexts, delayed childbearing may be associated with reduced lifetime earnings or interrupted career trajectories, particularly among women who re-enter the workforce after late pregnancies. Such economic strain may limit the resources available for healthy aging.

The strengths of our study include using multiple international datasets, enhancing generalizability and reliability. By examining both AMAC impact on frailty and the mediating role of life satisfaction, this analysis provides a perspective on long-term health among women with advanced maternal age at childbirth. Across three prospective cohorts, AMAC was linked to higher frailty incidence, with life satisfaction, an affective well-being indicator, partially mediating this relationship. These findings suggest that affective and psychosocial well-being represents a modifiable pathway for mitigating frailty risk in women with a history of AMAC. From a public health perspective, reproductive history may serve as an accessible life-course marker to help identify women who could benefit from earlier frailty screening, psychosocial risk assessment, and tailored behavioral support.

### Limitations of the study

In terms of study design, the observational nature of this research precludes definitive causal inference, and unobserved confounders may influence the observed relationships. Although we adjusted for national differences, residual confounding by social, economic, and cultural contexts—particularly regarding health management and social support—cannot be entirely ruled out. Furthermore, data attrition may compromise sample representativeness, and cross-cohort comparisons remain challenging given the heterogeneity in healthcare infrastructure, service access, and health behaviors across countries.

Regarding measurement, the FI, while constructed using a consistent deficit accumulation approach, relied on cohort-specific items due to differences in survey design. This between-cohort heterogeneity may introduce measurement variability and warrants caution in cross-cohort effect comparisons. Additionally, self-reported exposure and mediator variables may be subject to recall bias and misclassification. Finally, the ROC analysis should be interpreted cautiously, as the AUC captures the overall predictive performance of the multivariable model rather than the independent contribution of AMAC. Furthermore, although life satisfaction was treated as a mediator measured at baseline, its contemporaneous assessment with other baseline characteristics precludes us from definitively establishing the temporal direction of the association. Future research incorporating repeated measures of life satisfaction across the life course is needed to further disentangle this relationship.

## Resource availability

### Lead contact

Further information and requests for resources should be directed to and fulfilled by the lead contact, Xiaoyu Wang (wangxiaoyu@scau.edu.cn).

### Materials availability

This study did not generate new unique reagents.

### Data and code availability

• The CHARLS dataset was downloaded from https://charls.pku.edu.cn.
• The KLoSA dataset was downloaded from https://survey.keis.or.kr/eng/klosa/klosa01.jsp.
• The HRS dataset was downloaded from https://hrsdata.isr.umich.edu.
• This paper does not report original code.
• Any additional information required to reanalyze the data reported in this paper is available from the lead contact upon request.

## Acknowledgments

This work was funded by the 10.13039/501100001809National Natural Science Foundation of China (project no. 82401895).

## Author contributions

Concept and design, Z.-H.Z. and X.W.; acquisition, analysis, or interpretation of data, Z.-H.Z., W.T., and X.W.; drafting of the article, Z.-H.Z. and X.W.; critical revision of the article for important intellectual content, all authors; statistical analysis, X.W.; obtained funding, Z.-H.Z.; supervision, X.W. Z.-H.Z., W.T., and X.W. had full access to all of the data in the study, take responsibility for the integrity of the data and the accuracy of the data analysis, and contribute equally to this work.

## Declaration of interests

The authors declare no competing interests.

## STAR★Methods

### Key resources table

**Table:** 

REAGENT or RESOURCE	SOURCE	IDENTIFIER
**Deposited data**		

CHARLS cohort	https://charls.pku.edu.cn	IRB00001052-11015
KLoSA cohort	https://survey.keis.or.kr/eng/klosa/klosa01.jsp	336002
HRS cohort	https://hrsdata.isr.umich.edu	NIA U01AG009740

**Software and algorithms**		

Stata 16	https://www.stata.com/	StataCorp LLC
R 4.4	https://www.r-project.org/	The R Project

### Experimental model and study participant details

This study used data from three nationally representative longitudinal cohorts: CHARLS, KLoSA, and the HRS. Specifically, we used Wave 1 to Wave 5 for CHARLS, Wave 3 to Wave 8 for KLoSA, and Wave 10 to Wave 15 for HRS. For each cohort, baseline was defined as the first wave included in the present analysis: Wave 1 (2011) for CHARLS, Wave 3 (2010) for KLoSA, and Wave 10 (2010) for HRS. AMAC and baseline covariates were measured at the baseline wave, and participants were subsequently followed across later waves for incident frailty. The timelines of the three cohorts are shown in Figure S1. The study population was restricted to women aged ≥50 years. Participants were excluded if they reported implausible maternal age at childbirth (<21 or >55 years) or had missing data on frailty status and key covariates. Following these criteria, the final analytic samples comprised 7,060 participants from CHARLS, 4,103 from KLoSA, and 9,927 from HRS (Figure 1). The CHARLS cohort was approved by the Biomedical Ethics Review Committee of Peking University; the KLoSA cohort was approved by the National Statistical Office and Institutional Review Board of the Korea Centers for Disease Control and Prevention; and the HRS cohort was approved by the National Institute on Aging and the Social Security Administration. As the authors conducted secondary analysis of de-identified public-use data, no additional institutional review board approval was required.

### Method details

#### Baseline measurements

Baseline data collection encompassed demographic characteristics, socioeconomic status, and lifestyle factors, including age, educational attainment, smoking status, alcohol consumption, physical activity, and social engagement. Anthropometric measurements were recorded where available. Table 1 summarizes the baseline characteristics of the study population across the three cohorts.

#### Outcomes

Frailty was assessed using the frailty index, which aggregates age-related health deficits. Based on previous studies, frailty index construction involved items for HRS, CHARLS, and KLoSA,^39^^,^^40^ including cognition, chronic diseases, self-reported health, and functional disabilities (Table S1). All items, except cognition, were dichotomized (1 = deficit, 0 = no deficit); cognition was treated as a continuous variable, ranging from 0 to 1, with higher scores indicating poorer cognitive function. Frailty index was calculated by summing the item scores and dividing by the total number of items, resulting in a score range from 0 to 1, where higher values indicate greater frailty. In this study, a threshold of 0.25 was used to categorize participants as non-frail (frailty index <0.25) or frail (frailty index ≥0.25).^1^^,^^41^

#### Definitions

AMAC was defined as ≥35 years in CHARLS and KLoSA and as ≥40 years in HRS. Because the distribution of age at last childbirth differs across cohorts, we operationalized AMAC using a cohort-adapted cut-point. In CHARLS and KLoSA, age ≥35 years is a widely used threshold and provided adequate separation between exposure groups. In HRS, the age-35 threshold offered limited discrimination because the distribution was shifted toward older ages; as shown in the descriptive results (Table 2) and the five-category sensitivity analysis (Table S2), risk contrasts around age 35 were not evident, and the increased frailty risk was primarily concentrated among women with an age at last childbirth ≥40 years. Therefore, we used ≥40 years as the primary cut-point in HRS to capture the upper tail of the exposure distribution and to reduce attenuation due to exposure heterogeneity (i.e., dilution from grouping 35-39 years with ≥40 years). This approach aligns with trends in obstetric epidemiology, where the increasing prevalence of delayed childbearing has prompted some researchers to define a higher-risk “very advanced maternal age” group as women aged 40 or older (or even 45 or older), moving beyond the traditional ≥35-year cutoff.^42^^,^^43^

#### Mediator variable: Life satisfaction

Life satisfaction was measured as the mediator in this study. The assessment tools and measurement time points varied slightly across the three cohorts due to differences in their original survey designs.

In the CHARLS and HRS cohorts, life satisfaction was measured using a single-item, 5-point Likert scale. Participants were asked to rate their overall satisfaction with life. Responses were coded from 1 to 5, with higher scores indicating greater life satisfaction.

In the KLoSA cohort, life satisfaction was assessed using a visual analogue scale ranging from 0 to 100, where a higher score also reflected a higher level of life satisfaction.

Regarding the measurement time point, life satisfaction was collected at each survey wave. In our primary analytical model, we used the life satisfaction score reported at the baseline wave (Wave 1 for CHARLS in 2011, Wave 3 for KLoSA in 2010, and Wave 10 for HRS in 2010).

#### Exposure factors and primary outcome

The exposure of interest was AMAC. The primary outcome was incident frailty.

#### Covariates

Demographic characteristics (age, education, and marital status), health behaviors (smoking and drinking), lifestyle factors (social activity and physical activity), and socioeconomic indicators (employment and personal income) were included as covariates in multivariable models based on prior literature.^24^^,^^31^^,^^44^ Age was modeled as a continuous variable in the multivariable regression analyses, whereas an age-category variable was used for Kaplan–Meier analyses, subgroup analyses, and sensitivity analyses. Educational attainment was grouped into three categories according to the International Standard Classification of Education (ISCED) 1997: below high school, high school, and above high school. Marital status was dichotomized as married/partnered versus unmarried/other; the latter included individuals who were separated, divorced, widowed, or never married. Smoking status was classified as currently smoking versus not currently smoking. Alcohol use was defined based on any alcohol consumption in the past year (drinking vs. not drinking). Employment status was coded as unemployed versus currently working or retired, based on participants’ self-reported current employment. Social activity was categorized as yes versus no according to whether participants had engaged in any specified social activity during the past month. Physical activity was harmonized across cohorts using cohort-specific definitions of vigorous activity. In CHARLS and KLoSA, vigorous physical activity was defined as engaging in vigorous activity more than once per week; in HRS, it was defined as engaging in vigorous activity at least three times per week. Personal income was defined slightly differently across cohorts: in KLoSA, it reflects respondents’ after-tax wage and part-time earnings in the past year, whereas in CHARLS and HRS it represents total income in the past year. Following prior work,^45^ income was log-transformed to reduce skewness and improve model fit.

### Quantification and statistical analysis

First, we summarized the panel data using descriptive statistics to characterize the study samples across the three cohorts. Second, we used Kaplan-Meier survival curves to assess the unadjusted association between AMAC and frailty risk. We then fitted Cox proportional hazards models to estimate hazard ratios (HRs) and 95% confidence intervals (CIs) for the association between childbearing at an advanced age and incident frailty, with progressive adjustment for covariates (Table 2). Also, we verified the proportional hazards assumption for all Cox models by testing the significance of time-dependent covariates and examining Schoenfeld residuals. Restricted cubic spline (RCS) models were additionally fitted to examine potential non-linear dose-response relationships between maternal age and frailty risk. Receiver operating characteristic (ROC) curves and the area under the curve (AUC) were used to evaluate the discriminatory performance of models including AMAC for identifying frailty. We additionally fitted a covariate-only model excluding AMAC and compared its AUC with that of the corresponding model including AMAC, in order to assess the incremental discriminatory value of AMAC beyond conventional covariates.

To examine whether life satisfaction mediates the AMAC-frailty association, we performed mediation analysis using structural equation modeling (SEM). The mediation model was specified with three paths: (a) AMAC → life satisfaction, (b) life satisfaction → frailty index, and (c') direct effect of AMAC on frailty after including the mediator. All paths were adjusted for the same covariates as in the fully adjusted. The indirect effect was calculated as the product of path a and path b coefficients (a × b). Statistical significance was assessed using the product-of-coefficients method with the nlcom command in Stata 16.0, which provides point estimates, standard errors, and 95% confidence intervals based on the delta method.^46^ The proportion mediated was calculated as indirect effect divided by total effect [a × b/(c' + a × b)] when applicable. Analyses were conducted separately for each cohort.

Subgroup analyses were performed to evaluate potential effect modification across prespecified participant characteristics. We also conducted four sensitivity analysis to assess the robustness of the findings. To assess potential effect modification, we included multiplicative interaction terms (AMAC×subgroup variable) in the fully adjusted Cox models. The significance of interaction was evaluated using the Wald test for the interaction term (P-interaction). All analyses were conducted using Stata (version 16.0) and R (version 4.4.2). Two-sided P values <0.05 were considered statistically significant.

## References

[1] Zhang Z., Xu H., Zhang R., Yan Y., Ling X., Meng Y., Zhang X., Wang Y. (2025). Frailty and depressive symptoms in relation to cardiovascular disease risk in middle-aged and older adults. Nat. Commun..

[2] Dent E., Martin F.C., Bergman H., Woo J., Romero-Ortuno R., Walston J.D. (2019). Management of frailty: opportunities, challenges, and future directions. Lancet.

[3] Feng J., Chen X., Cai W., Zhou X., Zhang X. (2024). Association between inflammatory bowel disease and frailty: a two-sample Mendelian randomization study. Aging Clin Exp Res.

[4] Arantes F.S., Rosa Oliveira V., Leão A.K.M., Afonso J.P.R., Fonseca A.L., Fonseca D.R.P., Mello D., Costa I.P., Oliveira L.V.F., da Palma R.K. (2022). Heart rate variability: A biomarker of frailty in older adults?. Front Med (Lausanne).

[5] Ijaz N., Buta B., Xue Q.L., Mohess D.T., Bushan A., Tran H., Batchelor W., deFilippi C.R., Walston J.D., Bandeen-Roche K. (2022). Interventions for Frailty Among Older Adults With Cardiovascular Disease: JACC State-of-the-Art Review. J Am Coll Cardiol.

[6] Wright V.J., Schwartzman J.D., Itinoche R., Wittstein J. (2024). The musculoskeletal syndrome of menopause. Climacteric.

[7] Fried L.P., Tangen C.M., Walston J., Newman A.B., Hirsch C., Gottdiener J., Seeman T., Tracy R., Kop W.J., Burke G., McBurnie M.A. (2001). Frailty in older adults: evidence for a phenotype. J Gerontol A Biol Sci Med Sci.

[8] Matthews T.J., Hamilton B.E. (2009).

[9] Fitzpatrick K.E., Tuffnell D., Kurinczuk J.J., Knight M. (2017). Pregnancy at very advanced maternal age: a UK population-based cohort study. Bjog.

[10] Martin J.A., Kochanek K.D., Strobino D.M., Guyer B., MacDorman M.F. (2005). Annual summary of vital statistics--2003. Pediatrics.

[11] Cunningham F.G., Leveno K.J. (1995). Childbearing among older women--the message is cautiously optimistic. N. Engl. J. Med..

[12] Ye X., Baker P.N., Tong C. (2024). The updated understanding of advanced maternal age. Fundam. Res..

[13] Pinheiro R.L., Areia A.L., Mota Pinto A., Donato H. (2019). Advanced Maternal Age: Adverse Outcomes of Pregnancy, A Meta-Analysis. Acta Med Port.

[14] Lisonkova S., Potts J., Muraca G.M., Razaz N., Sabr Y., Chan W.S., Kramer M.S. (2017). Maternal age and severe maternal morbidity: A population-based retrospective cohort study. PLoS Med..

[15] Fan M., Wang D., Wu X., Gao W. (2024). Exploring the causal relationship between female reproductive traits and frailty: a two-sample mendelian randomization study. Front. Physiol..

[16] Ho V.W.T., Chua K.Y., Song X., Jin A., Koh W.P. (2024). Reproductive factors and risk of physical frailty among Chinese women living in Singapore. J. Nutr. Health Aging.

[17] Fujii K., Harada K., Kurita S., Morikawa M., Nishijima C., Kakita D., Shimada H. (2025). Life satisfaction as a protective factor against frailty among Japanese adults aged 60 and older: A cohort study. Maturitas.

[18] Fillit H., Butler R.N. (2009). The frailty identity crisis. J Am Geriatr Soc.

[19] Aasheim V., Waldenström U., Rasmussen S., Espehaug B., Schytt E. (2014). Satisfaction with life during pregnancy and early motherhood in first-time mothers of advanced age: a population-based longitudinal study. BMC Pregnancy Childbirth.

[20] Aasheim V., Waldenström U., Hjelmstedt A., Rasmussen S., Pettersson H., Schytt E. (2012). Associations between advanced maternal age and psychological distress in primiparous women, from early pregnancy to 18 months postpartum. Bjog.

[21] Bayrampour H., Heaman M. (2010). Advanced maternal age and the risk of cesarean birth: a systematic review. Birth.

[22] Waldenström U., Aasheim V., Nilsen A.B.V., Rasmussen S., Pettersson H.J., Shytt E. (2014). Adverse pregnancy outcomes related to advanced maternal age compared with smoking and being overweight. Obstet Gynecol.

[23] Nilsen A.B., Waldenström U., Hjelmstedt A., Rasmussen S., Schytt E. (2012). Characteristics of women who are pregnant with their first baby at an advanced age. Acta Obstet Gynecol Scand.

[24] Xian X., Xiang J., Cao S., Tang W., Wu Y., Li J., Ren S., Wang Y., Shen K. (2025). Associations between reproductive factors and frailty in middle-aged and older women: evidence from the China health and retirement longitudinal study. J. Health Popul. Nutr..

[25] Kojima G., Ogawa K., Iliffe S., Taniguchi Y., Walters K. (2020). Number of Pregnancies and Trajectory of Frailty Index: English Longitudinal Study of Ageing. J Am Med Dir Assoc.

[26] Taci D.Y., Yılmaz S., Arslan İ., Fidancı İ., Çelik M. (2023). The Evaluation of Frailty in the Elderly and Affecting Biopsychosocial Factors: A Cross-Sectional Observational Study. Iran. J. Public Health.

[27] Hajek A., König H.H. (2022). The association between the number of life births and certain frailty dimensions. Arch. Gerontol. Geriatr..

[28] Hao W., Wang Q., Yu R., Mishra S.R., Virani S.S., Shrestha N., Fu C., Zhu D. (2024). Reproductive factors and their association with physical and comprehensive frailty in middle-aged and older women: a large-scale population-based study. Hum Reprod Open.

[29] Chang T., Zhao Z., Liu X., Zhang Y., Liu X., Zhang Y., Lu M. (2025). The association between reproductive factors and frailty risk: a population-based analysis from the UK biobank. BMC Public Health.

[30] Kojima G., Taniguchi Y., Aoyama R., Urano T. (2022). Earlier menopause is associated with higher risk of incident frailty in community-dwelling older women in England. J Am Geriatr Soc.

[31] Gomes C.S., Pirkle C.M., Barbosa J.F.S., Vafaei A., Câmara S.M.A., Guerra R.O. (2018). Age at First Birth, Parity and History of Hysterectomy Are Associated to Frailty Status: Cross-Sectional Analysis from the International Mobility in Aging Study -Imias. J Cross Cult Gerontol.

[32] Guo H.J., Ye Y.L., Gao Y.F., Liu Z.H. (2024). Age at first birth is associated with the likelihood of frailty in middle-aged and older women: A population-based analysis from NHANES 1999-2018. Maturitas.

[33] Liu J., Wei W., Peng Q., Xue C., Yang S. (2022). The Roles of Life Satisfaction and Community Recreational Facilities in the Relationship between Loneliness and Depression in Older Adults. Clin. Gerontol..

[34] Smithson S.D., Greene N.H., Esakoff T.F. (2022). Pregnancy outcomes in very advanced maternal age women. Am. J. Obstet. Gynecol. MFM.

[35] Nes R.B., Czajkowski N.O., Røysamb E., Orstavik R.E., Tambs K., Reichborn-Kjennerud T. (2013). Major depression and life satisfaction: a population-based twin study. J Affect Disord.

[36] Liu H., Li D., Zhao X., Fang B., Zhang Q., Li T. (2021). Longitudinal Impact of Frailty States and Sleep Duration on Subsequent Depressive Symptoms of Older Adults. J Am Geriatr Soc.

[37] Lucas R.E., Clark A.E., Georgellis Y., Diener E. (2004). Unemployment alters the set point for life satisfaction. Psychol Sci.

[38] Gardner J., Oswald A.J. (2007). Money and mental wellbeing: a longitudinal study of medium-sized lottery wins. J. Health Econ..

[39] He D., Wang Z., Li J., Yu K., He Y., He X., Liu Y., Li Y., Fu R., Zhou D., Zhu Y. (2024). Changes in frailty and incident cardiovascular disease in three prospective cohorts. Eur. Heart J..

[40] Li L. (2024). Internet use and frailty in middle-aged and older adults: findings from developed and developing countries. Global Health.

[41] Fan J., Yu C., Guo Y., Bian Z., Sun Z., Yang L., Chen Y., Du H., Li Z., Lei Y. (2020). Frailty index and all-cause and cause-specific mortality in Chinese adults: a prospective cohort study. Lancet Public Health.

[42] Shrim A., Levin I., Mallozzi A., Brown R., Salama K., Gamzu R., Almog B. (2010). Does very advanced maternal age, with or without egg donation, really increase obstetric risk in a large tertiary center?. J. Perinat. Med..

[43] Jackson S., Hong C., Wang E.T., Alexander C., Gregory K.D., Pisarska M.D. (2015). Pregnancy outcomes in very advanced maternal age pregnancies: the impact of assisted reproductive technology. Fertil. Steril..

[44] Shin J.E., Han K.D., Shin J.C., Lee Y., Kim S.J. (2017). Association between maternal age at childbirth and metabolic syndrome in postmenopausal women: Korea National Health and Nutrition Examination Survey 2010 to 2012. Menopause.

[45] Vernon F., Morrow M., MaWhinney S., Coyle R., Coleman S., Ellison L., Zheng J.H., Bushman L., Kiser J.J., Galárraga O. (2020). Income Inequality Is Associated With Low Cumulative Antiretroviral Adherence in Persons With Human Immunodeficiency Virus. Open Forum Infect Dis.

[46] Huang S., MacKinnon D.P., Perrino T., Gallo C., Cruden G., Brown C.H. (2016). A Statistical Method for Synthesizing Mediation Analyses Using the Product of Coefficient Approach Across Multiple Trials. Stat Methods Appt.

