# Supplementary material for: Life satisfaction mediates advanced maternal age at childbirth and frailty across cohorts
Source: iScience. 2026 May 22;29(6):116023. doi: 10.1016/j.isci.2026.116023 (PMC13223979; doi:10.1016/j.isci.2026.116023)
Supplement: Document S1. Figures S1–S3 and Tables S1–S12 [file mmc1.pdf]

iScience, Volume *29*

Supplemental information

Life satisfaction mediates advanced maternal age at childbirth and frailty across cohorts

Zheng-Hui Zhao, Wenze Tian, and Xiaoyu Wang

Supplementary Files

Tables

Table S1. The items used to construct the frailty index in CHARLS, KLoSA and HRS.

No.	Description of the items			Cut-off value
	CHARLS (2011-2020)	HRS (2010-2021)	KLoSA (2010-2020)	
1	Self-reported, doctor-diagnosed hypertension			Yes=1, No=0
2	Self-reported, doctor-diagnosed diabetes			Yes=1, No=0
3	Self-reported, doctor-diagnosed cancer			Yes=1, No=0
4	Self-reported, doctor-diagnosed heart disease			Yes=1, No=0
5	Self-reported, doctor-diagnosed stroke			Yes=1, No=0
6	Self-reported, doctor-diagnosed arthritis			Yes=1, No=0
7	Self-reported, doctor-diagnosed chronic lung disease			Yes=1, No=0
10	Self-reported diagnosed any emotional, nervous, or psychiatric problems by doctors			Yes=1, No=0
11	Self-reported diagnosed memory-related disease, including Alzheimer's disease or dementia, organic brain senility, or other serious memory impairment			Yes=1, No=0
12	Self-reported eyesight for seeing thing up close			Blind, poor or fair=1, Good, very good or excellent=0
13	Self-reported hearing while wearing hearing aid or as usual			Deaf, poor or fair=1, Good, very good or excellent=0
14	Self-reported healthy status			Poor or fair=1, Good, very good or excellent=0
15	BADL: Any difficulty in dressing			Yes=1, No=0
16	BADL: Any difficulty in bathing or showering			Yes=1, No=0
17	BADL: Any difficulty in eating			Yes=1, No=0
18	BADL: Any difficulty in getting in/out bed			Yes=1, No=0
19	BADL: Any difficulty in using the toilet			Yes=1, No=0
20	IADL: Any difficulty in preparing meals			Yes=1, No=0
21	IADL: Any difficulty in shopping			Yes=1, No=0
22	IADL: Any difficulty in managing money			Yes=1, No=0
23	IADL: Any difficulty in taking medications			Yes=1, No=0
	CHARLS (2011-2020) , HRS (2010-2021)		KLoSA (2010-2020)	
24	Mobility: Any difficulty in walking 1 block		BADL: Any difficulty in brushb	Yes=1, No=0
25	Mobility: Any difficulty in getting up from a		BADL: Any difficulty in urinb	Yes=1, No=0

	chair after sitting for long periods		
26	Mobility: Any difficulty in climbing several flights of stairs without resting	IADL: Any difficulty in phoneb	Yes=1, No=0
27	Mobility: Any difficulty in stooping, kneeling or crouching	IADL: Any difficulty in transb	Yes=1, No=0
28	Mobility: Any difficulty in reaching arms above shoulder level	IADL: Any difficulty in gooutb	Yes=1, No=0
29	Mobility: Any difficulty in lifting or carrying weights over 10 pounds	IADL: Any difficulty in laundryb	Yes=1, No=0
30	Mobility: Any difficulty in picking up a coin from table	IADL: Any difficulty in groomb, housewkb	Yes=1, No= 0
31	Cognition: (total score - (immediate and delayed word recall + date naming)) / total score		Continuous, from 0 to 1, higher values correspond to worse cognitive function

Table S2. Comparison of baseline characteristics between included and excluded participants in the CHARLS cohort

Characteristic	Excluded (N=1,753)	Included (N=7,060)	P value
Maternal age			<0.001
<35 years	1,409 (81.35)	5,919 (84.91)	
≥35 years	323 (18.65)	1,052 (15.09)	
Age, mean (S.D.)	58.20 (10.32)	58.34 (10.49)	0.623
Education, n (%)			0.006
Below primary school	997 (56.91)	4,168 (59.15)	
Primary school	308 (17.58)	1,177 (16.71)	
Middle school	312 (17.81)	1,056 (14.99)	
High school and above	135 (7.71)	645 (9.15)	
Marital status, n (%)			0.004
Unmarried and others	231 (13.18)	1,124 (15.92)	
Married and partnered	1,522 (86.82)	5,936 (84.08)	
Smoking, n (%)			<0.001
No	1,666 (95.04)	6,450 (91.36)	
Yes	87 (4.96)	610 (8.64)	
Drinking, n (%)			0.213
No	1,522 (86.82)	6,048 (85.67)	
Yes	231 (13.18)	1,012 (14.33)	

Employment, n (%)			<0.001
Unemployed	1,626 (92.76)	6,256 (88.61)	
Working or retired	127 (7.24)	804 (11.39)	
Social activity, n (%)			<0.001
No	856 (48.83)	3,838 (54.36)	
Yes	897 (51.17)	3,222 (45.64)	
Physical activity, n (%)			0.018
No	1,157 (66.00)	4,446 (62.97)	
Yes	596 (34.00)	2,614 (37.03)	
Personal income (logarithm), mean (S.D.)	10.68 (2.01)	10.72 (1.96)	0.377

Note: **Included participants were those retained in the final Cox regression analysis, whereas excluded participants were female baseline participants who were not included in the final analysis after applying the predefined eligibility criteria. Continuous variables are presented as mean (S.D.), and categorical variables are presented as n (%). P values were derived from two-sample t tests for continuous variables and chi-square tests for categorical variables.**

Table S3. Comparison of baseline characteristics between included and excluded participants in the KLoSA cohort

**Characteristic**	**Excluded (N=406)**	**Included (N=4,103)**	**P value**
Maternal age			<0.001
<35	82 (43.62)	3,367 (82.07)	
≥35	106 (56.38)	736 (17.93)	
Age, mean (S.D.)	68.42 (11.50)	65.33 (10.84)	<0.001
Education, n (%)			0.003
Below high school	331 (81.53)	3,033 (73.92)	
High school	60 (14.78)	880 (21.45)	
Above high school	15 (3.69)	190 (4.63)	
Marital status, n (%)			<0.001
Unmarried and others	191(47.04)	1,360 (33.15)	
Married and partnered	215 [52.96)	2,743 (66.85)	
Smoking, n (%)			0.001
No	373 (91.87)	3,923 (95.61)	
Yes	33 (8.13)	180 (4.39)	
Drinking, n (%)			0.481

No	10 (2.46)	80 (1.95)	
Yes	396 (97.54)	4,023 (98.05)	
Employment, n (%)			0.141
Unemployed	79(19.46)	928 (22.65)	
Working or retired	327 (80.54)	3,169 (77.35)	
Social activity, n (%)			0.039
No	251 (61.82)	2,745 (66.90)	
Yes	155 (38.18)	1,358 (33.10)	
Physical activity, n (%)			0.004
0 times	309 (76.11)	2,841 (69.24)	
≥1 time	97 (23.89)	1,262 (30.76)	
Personal income (logarithm), mean(S.D)	0.71 (2.04)	1.13 (2.52)	0.001

***Note:* Included participants were those retained in the final Cox regression analysis, whereas excluded participants were female baseline participants who were not included in the final analysis after applying the predefined eligibility criteria. Continuous variables are presented as mean (S.D.), and categorical variables are presented as n (%). P values were derived from two-sample t tests for continuous variables and chi-square tests for categorical variables.**

Table S4. Comparison of baseline characteristics between included and excluded participants in the HRS cohort

Characteristic	Excluded (N=577)	Included (N=9,927)	P value
Maternal age			<0.001
<35 years	577 (100.00)	5,663 (57.04)	
≥35 years	0 (0.00)	4,264 (42.96)	
Age, mean (SD)	66.23 (11.58)	66.23 (11.66)	0.999
Education, n (%)			0.002
Below high school	97 (16.81)	1,880 (18.94)	
High school	339 (58.75)	6,209 (62.55)	
Above high school	141(24.44)	1,838 (18.51)	
Marital status, n (%)			0.891
Unmarried and others	254 (44.02)	4,399 (44.31)	
Married and partnered	323 (55.98)	5,528 (55.69)	
Smoking, n(%)			0.027
No	322 (55.81)	5,069 (51.06)	
Yes	255 (44.19)	4,858 (48.94)	

Drinking, n (%)			0.087
No	270 (46.79)	5,009 (50.46)	
Yes	307 (53.21)	4,918 (49.54)	
Employment, n (%)			0.834
Unemployed	353 (65.49)	5,976 (65.05)	
Working or retired	186 (34.51)	3,211 (34.95)	
Social activity, n (%)			0.194
No	411 (71.23)	6,815 (68.65)	
Yes	166 (28.77)	3,112 (31.35)	
Physical activity, n (%)			0.224
No	347 (60.14)	6,220 (62.66)	
Yes	230 (39.86)	3,707 (37.34)	
Personal income (logarithmic), mean (SD)	4.06 (5.00)	3.56 (4.84)	0.017

***Note:* Included participants were those retained in the final Cox regression analysis, whereas excluded participants were female baseline participants who were not included in the final analysis after applying the predefined eligibility criteria. Continuous variables are presented as mean (S.D.), and categorical variables are presented as n (%). P values were derived from two-sample t tests for continuous variables and chi-square tests for categorical variables.**

Table S5. Proportional hazards assumption test for Cox models (fully adjusted models)

Cohort	Global test x2	df	P-value
CHARLS	9.96	12	0.6199
KLoSA	20.53	12	0.0386
HRS	8.29	12	0.7621

Table S6. Comparison of AUCs for covariate-only models and models additionally including AMAC across CHARLS, HRS, and KLoSA

Cohort	Model	AUC	95% CI	P for comparison
CHARLS	Covariates only	0.6910	0.6751-0.7069	
CHARLS	Covariates + AMAC	0.6917	0.6759-0.7075	0.398
HRS	Covariates only	0.8406	0.8323-0.8489	
HRS	Covariates + AMAC	0.8403	0.8324-0.8490	0.459
KLoSA	Covariates only	0.8660	0.8482-0.8837	
KLOSA	Covariates + AMAC	0.8659	0.8481-0.8836	0.823

Table S7. Age at last childbirth (five categories) and incident frailty in CHARLS, KLoSA and HRS (Cox proportional hazards models 1–4).

Maternal	Model 1		Model 2		Model 3		Model 4	
**Age (years)**	**HR (95% CI)**	**P**	**HR (95% CI)**	**P**	**HR (95% CI)**	**P**	**HR (95% CI)**	**P**
**CHARLS**								
<25	0.91 (0.76–1.08)	0.286	1.02 (0.85–1.22)	0.820	1.02 (0.85–1.22)	0.863	1.04 (0.87–1.25)	0.668
25–29	0.89 (0.78–1.02)	0.091	0.96 (0.84–1.10)	0.552	0.96 (0.84–1.10)	0.560	0.98 (0.85–1.12)	0.731
30–34	1.00 (Ref.)	—	1.00 (Ref.)	—	1.00 (Ref.)	—	1.00 (Ref.)	—
35–39	1.35 (1.11–1.63)	0.002	1.19 (0.98–1.45)	0.075	1.18 (0.97–1.44)	0.090	1.18 (0.97–1.43)	0.104
≥40	1.59 (1.20–2.11)	0.001	1.28 (0.96–1.71)	0.092	1.28 (0.96–1.71)	0.089	1.26 (0.94–1.68)	0.120
**KLoSA**								
<25	0.47 (0.34–0.63)	<0.001	1.02 (0.75–1.40)	0.893	0.99 (0.72–1.35)	0.940	0.98 (0.71–1.34)	0.887
25–29	0.45 (0.37–0.55)	<0.001	0.74 (0.61–0.90)	0.003	0.74 (0.60–0.90)	0.002	0.76 (0.62–0.92)	0.006
30–34	1.00 (Ref.)	—	1.00 (Ref.)	—	1.00 (Ref.)	—	1.00 (Ref.)	—
35–39	1.62 (1.32–2.00)	<0.001	1.08 (0.88–1.34)	0.463	1.07 (0.86–1.32)	0.539	1.02 (0.83–1.27)	0.825
≥40	2.45 (1.80–3.35)	<0.001	1.21 (0.88–1.67)	0.234	1.19 (0.87–1.65)	0.276	1.07 (0.78–1.48)	0.668
**HRS**								
<25	1.18 (1.01–1.39)	0.042	1.17 (1.00–1.38)	0.051	1.15 (0.98–1.36)	0.084	1.12 (0.95–1.31)	0.184
25–29	1.14 (1.01–1.28)	0.029	1.16 (1.03–1.30)	0.015	1.15 (1.02–1.29)	0.020	1.11 (0.98–1.24)	0.094
30–34	1.00 (Ref.)	—	1.00 (Ref.)	—	1.00 (Ref.)	—	1.00 (Ref.)	—
35–39	1.09 (0.96–1.23)	0.184	1.04 (0.92–1.17)	0.560	1.03 (0.91–1.17)	0.606	1.04 (0.92–1.18)	0.491
≥40	1.31 (1.17–1.48)	<0.001	1.22 (1.08–1.38)	0.001	1.21 (1.07–1.36)	0.002	1.17 (1.03–1.32)	0.013

Notes: Exposure categories: <25, 25–29, 30–34 (reference), 35–39 and ≥40 years. Model 1 included the categorical exposure only. Model 2 adjusted for baseline age, education, and marital status. Model 3 further adjusted for smoking and drinking. Model 4 additionally adjusted for retirement/employment, social activity, physical activity, and log-transformed personal income. For Model 4 in CHARLS, P-overall (joint Wald test for categorical exposure) = 0.205; P-trend (modelled using category midpoints/medians) = 0.113. For Model 4 in KLoSA, P-overall = 0.046; P-trend = 0.040. For Model 4 in HRS, P-overall = 0.126; P-trend = 0.560.

Table S8. Association between AMAC and incident frailty after additional adjustment for parity.

	CHARLS		KLoSA		HRS*	
	HR (95%CI)	p	HR (95%CI)	p	HR (95%CI)	p
Model 5	1.13 (0.95, 1.33)	0.163	1.20 (0.98, 1.49)	0.083	1.35(1.08, 1.69)	0.008

Table S9. Sensitivity analyses excluding 2020 follow-up data for the association between AMAC and incident frailty in CHARLS, KLoSA, and HRS

	CHARLS		KLoSA		HRS*	
	**HR (95%CI)**	**p**	**HR (95%CI)**	**p**	**HR (95%CI)**	**p**
Model 1	1.52 (1.31, 1.76)	<0.001	2.96 (2.45, 3.58)	<0.001	1.22 (1.10, 1.36)	<0.001
Model 2	1.24 (1.05, 1.45)	0.009	1.27 (1.03, 1.57)	0.024	1.14 (1.02, 1.27)	0.019
Model 3	1.23 (1.05, 1.44)	0.012	1.26 (1.02, 1.56)	0.031	1.13 (1.02, 1.26)	0.022
Model 4	1.20 (1.02, 1.41)	0.024	1.18 (0.96, 1.55)	0.121	1.10 (0.99, 1.23)	0.076

Table S10. Interaction analyses between AMAC and frailty risk across subgroups in CHARLS cohort

Subgroups	HR (95% CI)	P-interaction
**Age (year)**		0.710
50-64	1.37 (1.04, 1.80)	
65-79	1.18 (0.86, 1.63)	
80 and older	1.29 (0.63, 2.64)	
**Education**		0.616
Below high school	1.18 (0.99, 1.40)	
High school	1.41 (0.93, 2.14)	
Above high school	1.61 (0.83, 3.14)	
**Marital status**		0.211
Unmarried/others	1.04 (0.78, 1.38)	
Married/partnered	1.28 (0.95, 1.73)	
**Smoking**		0.212
No	1.25 (1.06, 1.49)	
Yes	0.94 (0.67, 1.33)	
**Drinking**		0.800
No	1.19 (0.99, 1.43)	
Yes	1.25 (0.97, 1.60)	
**Employment**		0.247
Unemployed	1.59 (0.97, 2.61)	
Working/retired	1.17 (0.85, 1.62)	
**Social activity**		0.903
No	1.22 (0.97, 1.53)	
Yes	1.19 (0.96, 1.48)	
**Physical activity**		0.132
No	1.05 (0.81, 1.34)	
Yes	1.32 (1.05, 1.67)	

Note: All models adjusted for age, education, marital status, smoking, drinking, employment, social activity, physical activity, and log-transformed personal income. P-interaction values test the significance of interaction terms between AMAC and each subgroup variable.

Table S11. Interaction analyses between AMAC and frailty risk across subgroups in KLoSA cohort

Subgroups	HR (95% CI)	P-interaction
**Age (year)**		0.312
50-64	1.45 (0.52, 4.05)	
65-79	1.52 (0.54, 4.30)	
80 and older	1.12 (0.42, 2.99)	
**Education**		0.656
Below high school	0.97 (0.79, 1.20)	
High school	1.20 (0.79, 1.82)	
Above high school	1.91 (0.85, 4.31)	
**Marital status**		0.433
Unmarried/others	0.94 (0.73, 1.20)	
Married/partnered	1.08 (0.80, 1.46)	
**Smoking**		0.399
No	1.01 (0.83, 1.25)	
Yes	0.76 (0.52, 1.12)	
**Drinking**		0.601
No	0.77 (0.29, 2.03)	
Yes	1.00 (0.77, 1.30)	
**Employment**		0.830
Unemployed	0.84 (0.19, 3.74)	
Working/retired	0.99 (0.75, 1.31)	
**Social activity**		0.859
No	1.00 (0.79, 1.27)	
Yes	0.97 (0.73, 1.29)	
**Physical activity**		0.445
No	1.01 (0.82, 1.25)	
Yes	0.78 (0.54, 1.13)	

Note: All models adjusted for age, education, marital status, smoking, drinking, employment, social activity, physical activity, and log-transformed personal income. P-interaction values test the significance of interaction terms between AMAC and each subgroup variable.

Table S12. Interaction analyses between advanced maternal age at childbirth and frailty risk across subgroups in HRS cohort

Subgroups	HR (95% CI)	P-interaction
**Age (year)**		0.397
50-64	1.11 (0.91, 1.36)	
65-79	1.18 (0.97, 1.44)	
80 and older	1.01 (0.82, 1.24)	
**Education**		0.139
Below high school	0.93 (0.75, 1.15)	

High school	1.14 (0.96, 1.36)	
Above high school	1.28 (0.97, 1.70)	
**Marital status**		0.071
Unmarried/others	1.02 (0.89, 1.17)	
Married/partnered	1.23 (0.98, 1.54)	
**Smoking**		0.570
No	1.07 (0.93, 1.23)	
Yes	1.14 (0.99, 1.30)	
**Drinking**		0.042
No	1.02 (0.89, 1.16)	
Yes	1.27 (1.01, 1.59)	
**Employment**		0.418
Unemployed	1.18 (1.00, 1.37)	
Working/retired	1.03 (0.89, 1.19)	
**Social activity**		0.867
No	1.11 (0.99, 1.24)	
Yes	1.08 (0.96, 1.22)	
**Physical activity**		0.002
No	1.02 (0.91, 1.14)	
Yes	1.48 (1.23, 1.79)	

Note: All models adjusted for age, education, marital status, smoking, drinking, employment, social activity, physical activity, and log-transformed personal income. P-interaction values test the significance of interaction terms between AMAC and each subgroup variable.

Figures

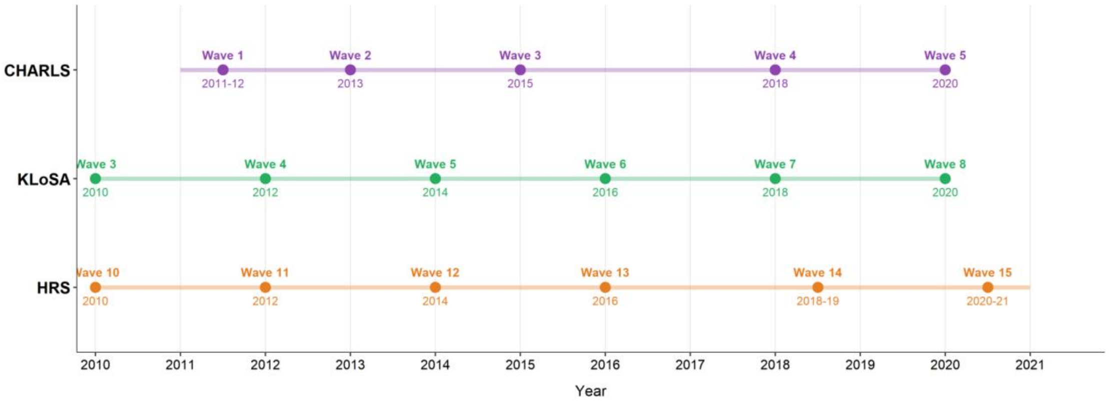

Figure S1. Timeline of the three longitudinal cohorts included in this study.

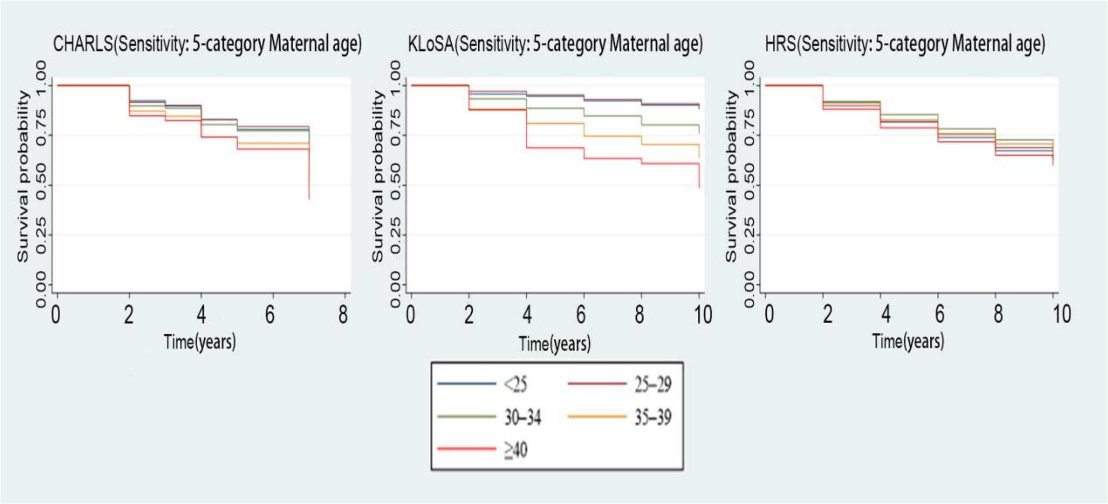

Figure S2. Kaplan-Meier survival curve of frailty risk by age at last childbirth (five categories) in CHARLS, KLoSA and HRS. Frailty risk: frailty status (non-frail or frail). Exposure categories were <25, 25 – 29, 30 – 34 (reference), 35 – 39 and ≥40 years. Log-rank test results across all three datasets indicate significant differences between groups (P < 0.001)

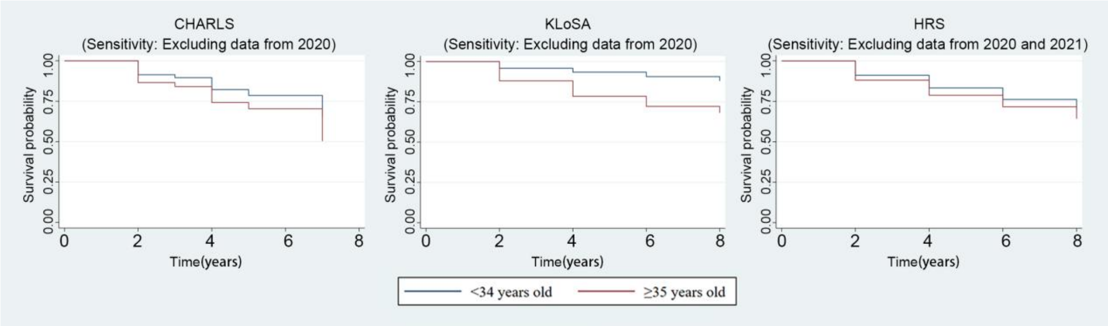

Figure S3. Kaplan – Meier curves for frailty-free survival according to AMAC status after excluding follow-up data from the year 2020 in CHARLS, KLoSA, and HRS
